# Identification of Core Prognosis-Related Candidate Genes in Chinese Gastric Cancer Population Based on Integrated Bioinformatics

**DOI:** 10.1155/2020/8859826

**Published:** 2020-12-11

**Authors:** Mengjun Li, Xinhai Wang, Jun Liu, Xiang Mao, Dongbing Li, Zhouyu Wang, Yifan Tang, Shuangjie Wu

**Affiliations:** ^1^Department of General Surgery, Huashan Hospital, Fudan University, Shanghai 200040, China; ^2^MyGene Diagnostics Co., Ltd., Guangzhou 510000, China

## Abstract

**Background:**

Gastric cancer (GC) is one of the leading causes of cancer-related mortality worldwide. There are great geographical differences in the incidence of GC, and somatic mutation rates of driver genes are also different. The present study is aimed at screening core prognosis-related candidate genes in Chinese gastric cancer population based on integrated bioinformatics for the early diagnosis and prognosis of GC.

**Methods:**

In the present study, the differentially expressed genes (DEGs) in GC were identified using four microarray datasets from the Gene Expression Omnibus (GEO) database. The samples of these datasets were all from China. Functional enrichment analysis of DEGs was conducted to evaluate the underlying molecular mechanisms involved in GC. Protein-protein interaction (PPI) network and cytoHubba were performed to determine hub genes associated with GC. Gene Expression Profiling Interactive Analysis (GEPIA) and Human Protein Atlas (HPA) were performed to validate the hub genes.

**Results:**

A total of 240 DEGs were obtained through the RRA method, including 80 upregulated genes and 160 downregulated genes. Upregulated genes were mainly enriched in extracellular matrix organization, extracellular matrix, and extracellular matrix structural constituent. The downregulated genes were mainly enriched in digestion, extracellular space, and oxidoreductase activity. The KEGG pathway enrichment analysis showed that the upregulated genes were mainly associated with ECM-receptor interaction, focal adhesion, and PI3K-Akt signaling pathway. And downregulated genes were mainly associated with the metabolism of xenobiotics by cytochrome P450, metabolic pathways, and gastric acid secretion. The transcriptional and translational expression levels of the genes including *COL1A1*, *COL5A2*, *COL12A1*, and *VCAN* were higher in GC tissues than normal tissues.

**Conclusion:**

A total of four genes including *COL1A1*, *COL5A2*, *COL12A1*, and *VCAN* were considered potential GC biomarkers in the Chinese population. And ECM-receptor interaction, focal adhesion, and PI3K-Akt signaling pathway were revealed to be important mechanisms of GC. Our findings provide novel insights into the occurrence and progression of GC in the Chinese population.

## 1. Introduction

Gastric cancer (GC) is one of the most common malignancies worldwide, and it is the third leading cause of cancer-related death [1]. The incidence of GC is the highest among East Asians [2]. GC is a multifactorial disease, where many factors can influence its development, both environmental and genetic [3, 4]. According to reports, certain lifestyles increase the risk of stomach cancer, including smoking, obesity, high salt and salted food intake, and low intake of fruits and vegetables [5].

According to most large clinical studies, patients have a poor prognosis, with a five-year survival rate of less than 25% and an average overall survival (OS) of 7 to 10 months after diagnosis [6, 7]. The clinical outcome of GC depends on the tumor stage at the time of diagnosis. As GC symptoms usually only appear in the late stage, many GC patients have advanced disease after a definite diagnosis [8]. Surgery, chemotherapy, and radiation therapy are the most common treatments. For patients with early GC, radical gastrectomy is the preferred method for the treatment of localized GC, but the recurrence rate is still high [9]. For patients whose tumor cannot be surgically removed or have advanced metastases, chemotherapy is the most important treatment [10]. However, because of inherent or acquired resistance, patients with GC often have poor or no response to chemotherapy [11]. Immune checkpoint inhibitors (ICIs) are currently being studied as the first-line treatment. In addition, new combinations of ICIs and targeted drugs are being evaluated in clinical trials [12]. Despite advances in treatment, the clinical outcome of patients with advanced GC is still poor. There are great geographical differences in the incidence of GC, and somatic mutation rates of driver genes are also different [13]. In the Chinese population, molecular markers for GC need to be extended. Early detection and treatment are critical to reduce GC mortality [14]. In the era of targeted therapy, mutational analysis of cancer is a key aspect of making treatment decisions [15]. Therefore, it is crucial to identify a sensitive and specific biomarker that can predict the prognosis of GC and be a target for GC treatment in China. The Gene Expression Omnibus (GEO) database (http://www.ncbi.nlm.nih.gov/geo/) was used for the bioinformatics data mining of gene expression profiles [16]. At present, DNA microarray and bioinformatics analysis methods were used to identify potential biomarkers that affect the development of diseases in studies [17].

In the present study, the differentially expressed genes (DEGs) in GC were identified using four microarray datasets from the GEO database. Subsequently, Gene Ontology (GO) annotation and Kyoto Encyclopedia of Genes and Genomes (KEGG) pathway enrichment analyses were conducted to evaluate the underlying molecular mechanisms involved in carcinogenesis and tumor progression. Protein-protein interaction (PPI) network and cytoHubba were performed to determine hub genes associated with GC. Survival analyses of the screened hub genes were carried out using Gene Expression Profiling Interactive Analysis (GEPIA). The expression levels of the identified hub genes were validated based on GEPIA and Human Protein Atlas (HPA) online databases. Our study will provide some useful biomarkers which could be promising and effective targets for diagnosis and prognosis of GC.

## 2. Materials and Methods

### 2.1. Microarray Data

The gene expression profile data (GSE118916, GSE54129, GSE79973, and GSE19826) for gastric cancer were downloaded from the GEO database (https://www.ncbi.nlm.nih.gov/geo/). The selection criteria for these datasets were as follows: (i) the samples in each dataset were from China, (ii) included datasets must include paired GC and normal control tissues, and (iii) sample size of each group must be ≥10. GSE54129, GSE79973, and GSE19826 were based on the GPL570 platform [(HG-U133_Plus_2) Affymetrix Human Genome U133 Plus 2.0 Array], and GSE118916 was based on GPL15207 platform [(PrimeView) Affymetrix Human Gene Expression Array]. The dataset information is shown in Table 1. Four datasets totally included 148 GC tissues and 58 normal gastric tissues.

### 2.2. Data Preprocessing and Identification of DEGs

R language command was used to convert the gene probe IDs in the matrix files to the gene symbols in the platform files to obtain a matrix file containing the international standard gene name. Each dataset was then normalized using the limma R package. All gene expression data were subjected to log2 transformation. The limma R package was used to screen for DEGs in each dataset [18]. Gene integration for the DEGs screened from the four datasets was executed using the RobustRankAggreg (RRA) package based on a robust rank aggregation method [19]. The RRA method was based on the assumption that all genes were unordered in each list. Genes that met the specific cut-off criteria of adjusted *P* value < 0.05 and logFC | >1.0 were regarded as DEGs.

### 2.3. GO Annotations and KEGG Pathway Enrichment Analyses of DEGs

DAVID 6.8 (https://david.ncifcrf.gov/) was performed to analyze the enrichment of GO and KEGG pathways of DEGs. The results were considered statistically significant if *P* < 0.05. Then, the R ggplot2 package was performed to visualize the significant GO terms and KEGG pathways.

### 2.4. PPI Network Constructions and Analysis of Modules

Protein-protein interactions among overlapping DEGs were identified via the STRING database, and genes with the combined score 0.4 were selected to construct the PPI network [20]. The PPI network was visualized and analyzed by Cytoscape 3.8.0, a practical open-source software tool that visually explores bimolecular interaction networks composed of proteins, genes, and other types of interaction. Five methods in plug-in cytoHubba were used to select the key genes in PPI, namely, EPC (edge percolated component), MCC (maximal clique centrality), MNC (maximal neighborhood component), degree (node connect degree), and closeness (node connect closeness). Top 20 genes in each method were selected, and then, the intersection was taken to get the key genes in the PPI analysis [21]. Hub network modules were captured with the help of the Cytoscape plug-in Molecular Complex Detection (MCODE) with parameters degree cutoff = 2, node score cutoff = 0.2, and *K* − core = 2 [22].

### 2.5. Survival Analyses and RNA Sequencing Expression of Hub Genes

To validate the expression of the key DEGs, the Gene Expression Profiling Interactive Analysis (GEPIA) website (http://gepia2.cancer-pku.cn/#index) was applied to analyze the data of RNA sequencing expression based on thousands of samples from the GTEx projects and TCGA [23]. The association between overall survival (OS) and the genes expressed in GC patients was determined using GEPIA. The lower and upper 50% of gene expression were set as the standard for analysis. Log-rank test results with *P* < 0.05 were regarded as statistically significant. Besides, the GEPIA was employed to visualize the mRNA expression of hub genes in tumors and normal samples.

### 2.6. Exploration of the Protein Levels of Hub Genes in the Human Protein Atlas Database

The Human Protein Atlas (HPA) database (https://www.proteinatlas.org/) is an free online database that provides abundant transcriptome and proteome data on human normal or pathological tissues through RNA sequence analysis and immunohistochemical analysis. In the present study, the protein expression and distribution of hub genes were investigated in GC tissues and compared normal tissues in HPA [24].

## 3. Results

### 3.1. Identification of DEGs in GC

**Figure 1:**
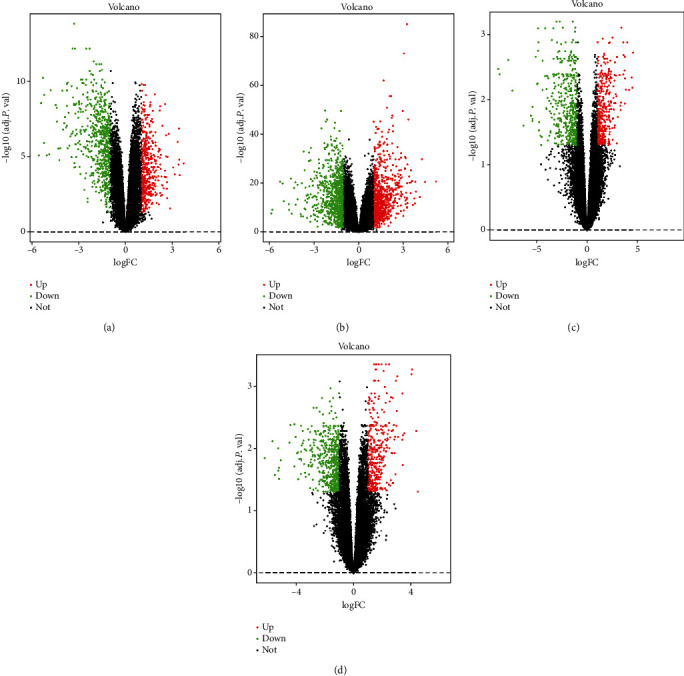
The DEGs of the four datasets. The DEGs in (a) GSE118916, (b) GSE54129, (c) GSE79973, and (d) GSE19826 datasets. The red dots represent upregulated genes according to adjustment *P* < 0.05 and log fold − change > 1; the green dots represent downregulated genes after adjustment *P* < 0.05 and log fold − change > 1; and the black dots represent genes with no significant difference in expression.

**Figure 2:**
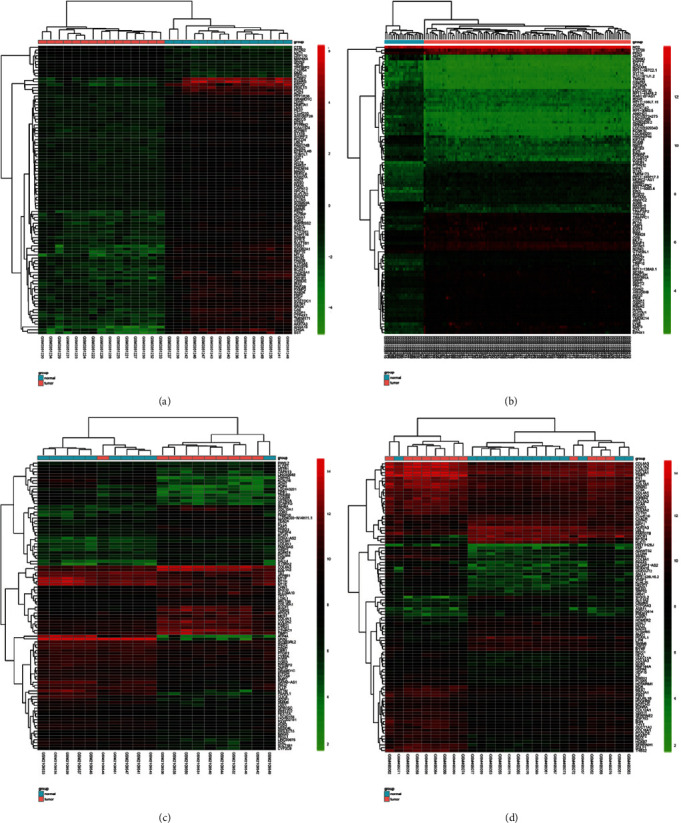
Cluster heat maps of the top 100 DEGs in four datasets. The heat map of the top DEGs in (a) GSE118916, (b) GSE54129, (c) GSE79973, and (d) GSE19826 datasets. Red indicates relatively upregulated gene expression; green indicates relatively downregulated gene expression; black indicates no significant change in gene expression; and gray scale indicates that the signal strength is not high enough to detect.

**Figure 3:**
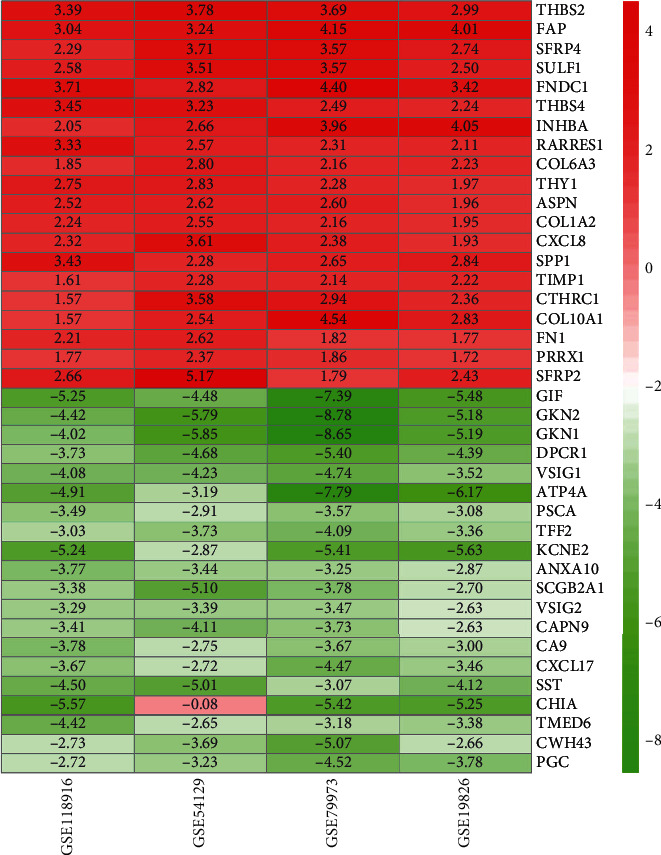
The top 20 up- and downregulated genes in integrated datasets. The abscissa represents the GEO datasets, and the ordinate represents the gene name. The red represents log FC > 0; the pink represents log FC is slightly less than 0; and the green represents log FC < 0.

The GC chip expression datasets GSE118916, GSE54129, GSE79973, and GSE19826 were normalized, and the results are shown in Figure S1. The GSE118916 dataset contained 1143 differential genes, including 511 upregulated genes and 632 downregulated genes. The GSE54129 dataset contained 1793 differential genes, including 894 upregulated genes and 899 downregulated genes. The GSE79973 dataset contained 857 differential genes, including 410 upregulated genes and 447 downregulated genes. In addition, the GSE19826 dataset contained differential genes, including 387 upregulated genes and 504 downregulated genes. The DEGs of the four datasets are shown in Figure 1, and the cluster heat map of the top 100 genes is shown in Figure 2. The batch effect can be eliminated by RRA method. A total of 240 DEGs were obtained through the RRA method, including 80 upregulated genes and 160 downregulated genes (Table S1). The top 20 up- and downregulated genes after the integrated analysis are displayed in Figure 3.

### 3.2. Functional Enrichment Analyses

**Figure 4:**
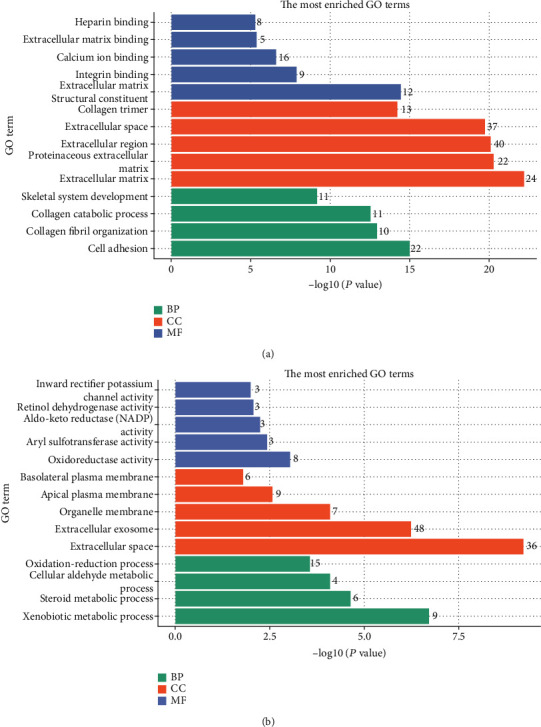
The results of GO analysis of upregulated genes (a) and downregulated genes (b). BP: biological process; CC: cellular component; MF: molecular function.

**Figure 5:**
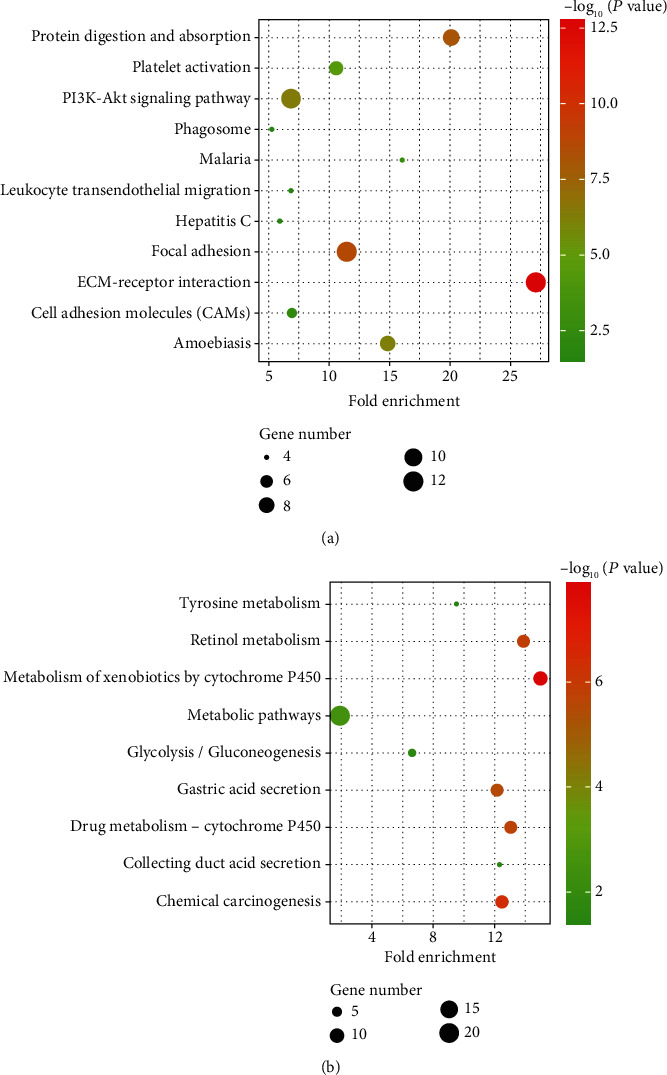
KEGG pathway enrichment analyses of DEGs. (a) The KEGG pathway enrichment analysis of upregulated genes. (b) The KEGG pathway enrichment analysis of downregulated genes.

GO functional analysis of integrated differential genes was divided into three parts: biological process (BP), cellular component (CC), and molecular function (MF). The top 15 GO terms of BP, CC, and MF of upregulated and downregulated genes are shown in Table 2. The results of 15 GO analyses of upregulated genes and downregulated genes are shown in Figure 4. Upregulated genes were mainly enriched in extracellular matrix organization, extracellular matrix, and extracellular matrix structural constituent. The downregulated genes were mainly enriched in digestion, extracellular space, and oxidoreductase activity. According to the KEGG pathway enrichment analysis, the upregulated genes were mainly associated with ECM-receptor interaction, focal adhesion, and PI3K-Akt signaling pathway (Figure 5(a)). And downregulated genes were mainly associated with the metabolism of xenobiotics by cytochrome P450, metabolic pathways, and gastric acid secretion (Figure 5(b)).

### 3.3. PPI Network and Module Analyses

**Figure 6:**
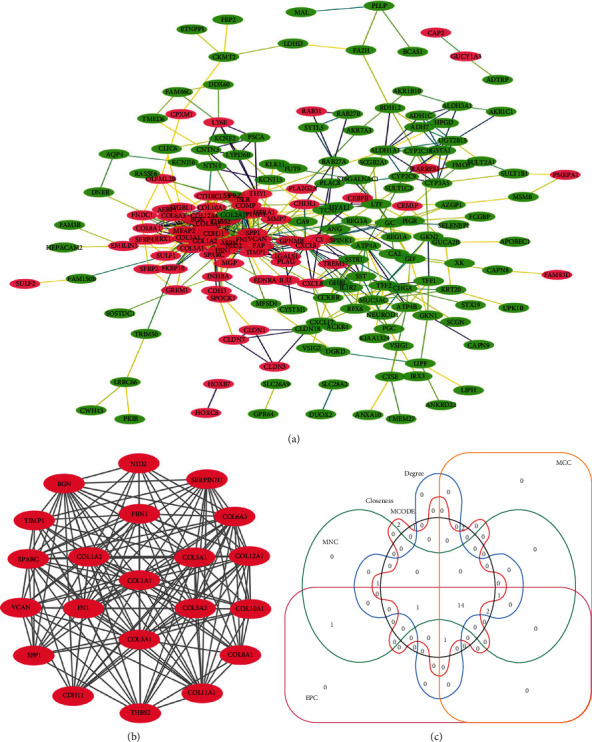
Construction of PPI network and module analysis. (a) The PPI network of DEGs. The red circles meant upregulated DEGs and green circles meant downregulated DEGs. (b) Module 1 of PPI network. (c) Venn diagram of overlapping DEGs common to EPC, MCC, MNC, degree, closeness, and MCODE. EPC: edge percolated component; MCC: maximal clique centrality; MNC: maximal neighborhood component; degree: node connect a degree; closeness: node connect closeness.

The PPI network was constructed by Cytoscape based on the STRING database, consisting of 193 nodes and 615 edges (Figure 6(a)). The genes that scored in the top 20 by five methods are shown in Table 3. MCODE in Cytoscape was used to perform module analysis. The most important module (Module 1) was selected, as shown in Figure 6(b). This model included 21 nodes and 164 edges. Remarkably, genes in this module were all upregulated. We found that most of genes in the top 20 genes in five methods were in Module 1 (Figure 6(c)). There were 14 genes, including *BGN*, *CDH11*, *COL12A1*, *COL1A1*, *COL1A2*, *COL3A1*, *COL5A1*, *COL5A2*, *FBN1*, *FN1*, *SPARC*, *THBS2*, *TIMP1*, and *VCAN*. KEGG pathway enrichment analysis of the 14 genes was performed using the DAVID website (Table S2). The results showed that the key genes were mainly enriched in ECM-receptor interaction, focal adhesion, and PI3K-Akt signaling pathway.

### 3.4. Analysis of Hub Genes in the GEPIA

**Figure 7:**
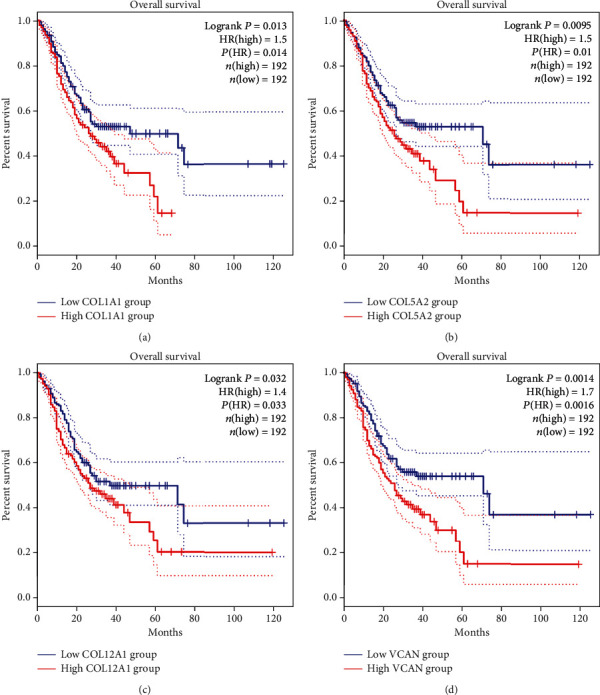
The OS (overall survival) analysis of hub genes by GEPIA. Four hub genes were found to be associated with the prognosis of gastric cancer patients. (a) *COL1A1*. (b) *COL5A2*. (c) *COL12A1*. (d) *VCAN*.

**Figure 8:**
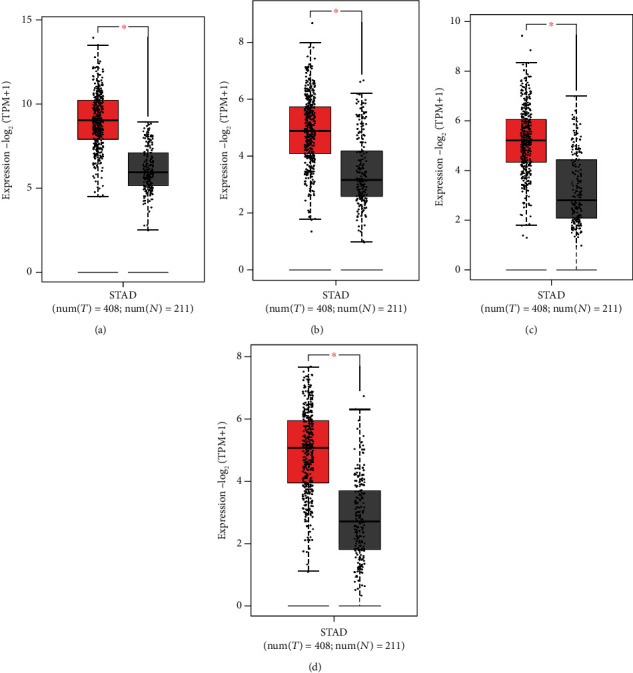
Validation of the mRNA expression levels of (a) *COL1A1*, (b) *COL5A2*, (c) *COL12A1*, and (d) *VCAN* in GC tissues and normal stomach tissues using GEPIA. The red box represents GC samples (408), and the gray box represents normal samples (211). GC: gastric cancer; STAD: stomach adenocarcinoma.

As shown in Figure 7, high expression levels of *COL1A1*, *COL5A2*, *COL12A1*, and *VCAN* in patients with GC were associated with poor OS. Besides, the expression levels of four genes in GC tissues were significantly higher than in normal tissues (Figure 8).

### 3.5. Validation of Hub Genes via the HPA

**Figure 9:**
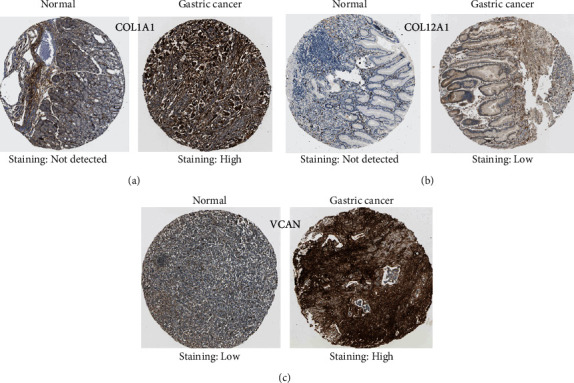
Representative immunohistochemistry images of (a) COL1A1, (b) COL12A1, and (c) VCAN in GC and noncancerous stomach tissues derived from the HPA database. HPA: Human Protein Atlas.

The protein expression levels of these hub genes in GC were explored using the HPA database (Figure 9). The protein levels of COL1A1 and COL12A1 were not expressed in normal stomach tissues, whereas the high protein expression levels of COL1A1 and low protein expression levels of COL12A1 were observed in GC tissues. The low protein expression levels of VCAN were observed in normal stomach tissues, while high protein expression levels of VCAN were observed in GC tissues. There was no pathological map of COL5A2 expression in GC in the HPA database. In summary, the present results indicated that the transcriptional and translational expression levels of the hub genes were overexpressed in patients with GC.

## 4. Discussion

Despite significant advances in GC treatment protocols, the underlying mechanism of GC development and progression is still unclear, and more cancer-related molecules have yet to be discovered. Bioinformatics analysis has been playing crucial roles in cancer study [25]. Among various bioinformatics strategies, DNA microarray gene expression profiling has been widely used to explore DEGs involved in tumorigenesis, diagnosis, and treatment [26]. At present, most of the GEO datasets used for CRC research are from different countries [27]. For the first time, we analyzed 4 GEO datasets from the Chinese gastric cancer population and used bioinformatics to discover possible biomarkers of GC.

In the present study, 240 DEGs containing 80 upregulated genes and 160 downregulated genes were screened and integrated from four GEO datasets. The 240 integrated DEGs were then subjected to BP, CC, and MF enrichment analyses. Upregulated genes were mainly enriched in extracellular matrix organization, extracellular matrix, and extracellular matrix structural constituent. The downregulated genes were mainly enriched in digestion, extracellular space, and oxidoreductase activity. These results indicated that DEGs were mainly involved in the progression of GC through extracellular matrix. The extracellular matrix is a key component exerting an active effect in all the hallmarks of cancer [28]. KEGG pathway analysis demonstrated that the upregulated genes were mainly enriched in ECM-receptor interaction, focal adhesion, and PI3K-Akt signaling pathway. The downregulated genes were mainly associated with the metabolism of xenobiotics by cytochrome P450, metabolic pathways, and gastric acid secretion. ECM-receptor interaction and focal adhesion have been shown to be important components of tumorigenesis and cancer progression [29, 30]. The PI3K-Akt pathway is widely distributed in various cells and is known to regulate cell behavior, protein synthesis, and angiogenesis [31]. The disorder of the PI3K-Akt pathway may trigger the occurrence and development of cancer [32]. Studies have found that cytochrome P450 family genes were involved in the development of gastric adenocarcinoma through the metabolism of xenobiotics by cytochrome P450 [33]. Genetic variations of gastric acid secretion pathway genes are associated with the risk of GC [34]. Studying these pathways will help to elucidate the underlying mechanism of GC development and progression.

Through the PPI network construction and analysis of modules, we identified the following 14 hub genes: *BGN*, *CDH11*, *COL12A1*, *COL1A1*, *COL1A2*, *COL3A1*, *COL5A1*, *COL5A2*, *FBN1*, *FN1*, *SPARC*, *THBS2*, *TIMP1*, and *VCAN*. The GEPIA and HPA were applied for further validation of the expression level of these genes. Finally, we identified 4 important genes (*COL1A1*, *COL5A2*, *COL12A1*, and *VCAN*). The expression levels of the four genes in GC tissues were significantly higher than in normal tissues. The genes *COL1A1* and *COL5A2* belong to the collagen gene family, which participates in the formation of collagen in extracellular matrix proteins [35]. As a key structural component of ECM, collagen has been found to be overexpressed in a variety of cancers, providing a rigid matrix that promotes tumor growth [36]. Studies have reported that *COL1A1* and *COL1A2* were generally upregulated in GC and were associated with invasion and metastasis [37]. *COL5A2* has previously been found to be associated with the pathological processes of GC [38]. Although bioinformatics analysis has suggested that COL5A2 is a candidate GC biomarker, its precise regulatory mechanism is still unclear [39]. *COL1A1* and *COL5A2* are members of three important pathways that upregulated gene enrichment. *COL12A1*, a gene encoding collagen type XII alpha 1 chain, is a typical collagen-organizer molecule involved in collagen cross-linking in the cancer microenvironment [40]. The expression of COL12A1 in GC tissues increased significantly, and the elevated COL12A1 protein level was positively correlated with aggressive clinical features [41]. *VCAN* is a chondroitin sulfate proteoglycan. A study showed that the upregulation of *VCAN* promoted the migration and invasion of ovarian cancer cells by activating the NF-*κ*B signaling pathway [42]. Wnt and chemokine signaling pathways could be key regulators of *VCAN* expression in GC [43].

The present study has certain limitations such as the sample size for the RNA-Seq experiments and lack of validation in tumor tissues. Besides, the characteristic details (such as gender, age, race, tumor grade, and staging) were not taken into account in our research.

## 5. Conclusion

In the present study, 240 differentially expressed genes were identified in the GEO datasets from the Chinese GC population. Four of them (*COL1A1*, *COL5A2*, *COL12A1*, and *VCAN*) were considered potential GC biomarkers. In the database, the expression levels of four genes in GC tissues were significantly higher than in normal tissues. ECM-receptor interaction, focal adhesion, and PI3K-Akt signaling pathway were revealed to be important mechanisms of GC. The present study provided novel insights into the occurrence and progression of GC in the Chinese population. However, the diagnostic and prognostic valuse of these genes require further validation.

## Figures and Tables

**Table 1:** Details of the GEO gastric cancer data.

Dataset	Platform	Number of samples (tumor/control)
GSE118916	GPL15207 [PrimeView] Affymetrix Human Gene Expression Array	30 (15/15)
GSE54129	GPL570 [HG-U133_Plus_2] Affymetrix Human Genome U133 Plus 2.0 Array	132 (111/21)
GSE79973	GPL570 [HG-U133_Plus_2] Affymetrix Human Genome U133 Plus 2.0 Array	20 (10/10)
GSE19826	GPL570 [HG-U133_Plus_2] Affymetrix Human Genome U133 Plus 2.0 Array	27 (12/15)

GEO: Gene Expression Omnibus.

**Table 2:** The top 15 GO terms of BP, CC, and MF of upregulated and downregulated genes.

Category	(A) The top 15 enriched GO terms of upregulated genes		
	Term	Count	*P* value
BP	Extracellular matrix organization	20	2.12*E*-20
BP	Cell adhesion	22	9.65*E*-16
BP	Collagen fibril organization	10	1.05*E*-13
BP	Collagen catabolic process	11	2.79*E*-13
BP	Skeletal system development	11	6.47*E*-10
CC	Extracellular matrix	24	6.15*E*-23
CC	Proteinaceous extracellular matrix	22	5.02*E*-21
CC	Extracellular region	40	7.52*E*-21
CC	Extracellular space	37	1.93*E*-20
CC	Collagen trimer	13	5.06*E*-15
MF	Extracellular matrix structural constituent	12	3.51*E*-15
MF	Integrity binding	9	1.32*E*-08
MF	Calcium ion binding	16	2.27*E*-07
MF	Extracellular matrix binding	5	4.00*E*-06
MF	Heparin binding	8	4.82*E*-06

Category	(B) The top 15 enriched GO terms of downregulated genes		
	Term	Count	*P* value

BP	Digestion	14	2.25*E*-15
BP	Xenobiotic metabolic process	9	1.95*E*-07
BP	Steroid metabolic process	6	2.27*E*-05
BP	Cellular aldehyde metabolic process	4	7.82*E*-05
BP	Oxidation-reduction process	15	2.75*E*-04
CC	Extracellular space	36	6.18*E*-10
CC	Extracellular exosome	48	5.83*E*-07
CC	Organelle membrane	7	7.91*E*-05
CC	Apical plasma membrane	9	0.002747
CC	Basolateral plasma membrane	6	0.016259
MF	Oxidoreductase activity	8	9.13*E*-04
MF	Aryl sulfotransferase activity	3	0.003693
MF	Aldo-keto reductase (NADP) activity	3	0.005787
MF	Retinol dehydrogenase activity	3	0.008306
MF	Inward rectifier potassium channel activity	3	0.010212

BP: biological process; CC: cellular component; MF: molecular function.

**Table 3:** The genes that scored in the top 20 by EPC, MCC, MNC, degree, and closeness.

Category	Rank methods in cytoHubba				
	EPC	MCC	MNC	Degree	Closeness
1	COL1A2	COL1A1	COL1A1	FN1	FN1
2	COL1A1	COL1A2	COL3A1	COL1A1	COL1A1
3	COL5A2	COL3A1	FN1	COL3A1	COL3A1
4	COL3A1	COL5A1	COL1A2	COL1A2	COL1A2
5	FN1	COL5A2	BGN	BGN	CXCL8
6	FBN1	COL11A1	COL5A2	COL5A2	SPP1
7	COL5A1	FN1	FBN1	FBN1	BGN
8	BGN	BGN	TIMP1	TIMP1	TIMP1
9	VCAN	COL6A3	THBS2	THBS2	FBN1
10	TIMP1	FBN1	SPARC	SPARC	VCAN
11	SERPINH1	SPARC	COL5A1	VCAN	COL5A2
12	CDH11	COL12A1	SPP1	SPP1	SPARC
13	THBS2	THBS2	COL6A3	COL5A1	THY1
14	COL2A1	COL2A1	VCAN	COL6A3	COL2A1
15	COL12A1	SERPINH1	COL12A1	CXCL8	THBS2
16	SPP1	VCAN	COL2A1	COL12A1	C3
17	COL11A1	COL10A1	CDH11	COL2A1	CDH11
18	SPARC	COL8A1	CXCL8	CDH11	COL5A1
19	COL6A3	CDH11	COL11A1	COL11A1	COL12A1
20	ASPN	TIMP1	ASPN	SERPINH1	SERPINH1

EPC: edge percolated component; MCC: maximal clique centrality; MNC: maximal neighborhood component; Degree: node connect degree; Closeness: node connect closeness.

## Data Availability

The data used to support the findings of this study are available from the corresponding author upon request.

## Supplementary Materials

Supplementary Materials Figure S1: normalization of gene expression. (A, B) Normalization of the GSE118916 dataset. (C, D) Normalization of the GSE54129 dataset. (E, F) Normalization of the GSE79973 dataset. (G, H) Normalization of the GSE19826 dataset. Blue represents data before normalization, and red represents data after normalization. Table S1: the integrated DEGs in gastric cancer. Table S2: the KEGG analysis of the 14 genes.
