# Supplementary material for: Identification of Core Prognosis-Related Candidate Genes in Chinese Gastric Cancer Population Based on Integrated Bioinformatics
Source: Biomed Res Int. 2020 Dec 11;2020:8859826. doi: 10.1155/2020/8859826 (PMC7748906; doi:10.1155/2020/8859826)
Supplement: Supplementary Materials — Figure S1: normalization of gene expression. (A, B) Normalization of the GSE118916 dataset. (C, D) Normalization of the GSE54129 dataset. (E, F) Normalization of the GSE79973 dataset. (G, H) Normalization of the GSE19826 dataset. Blue represents data before normalization, and red represents data after normalization. Table S1: the integrated DEGs in gastric cancer. Table S2: the KEGG analysis of the 14 genes. [file 8859826.f1.docx]

**Supplementary Figures and Tables**


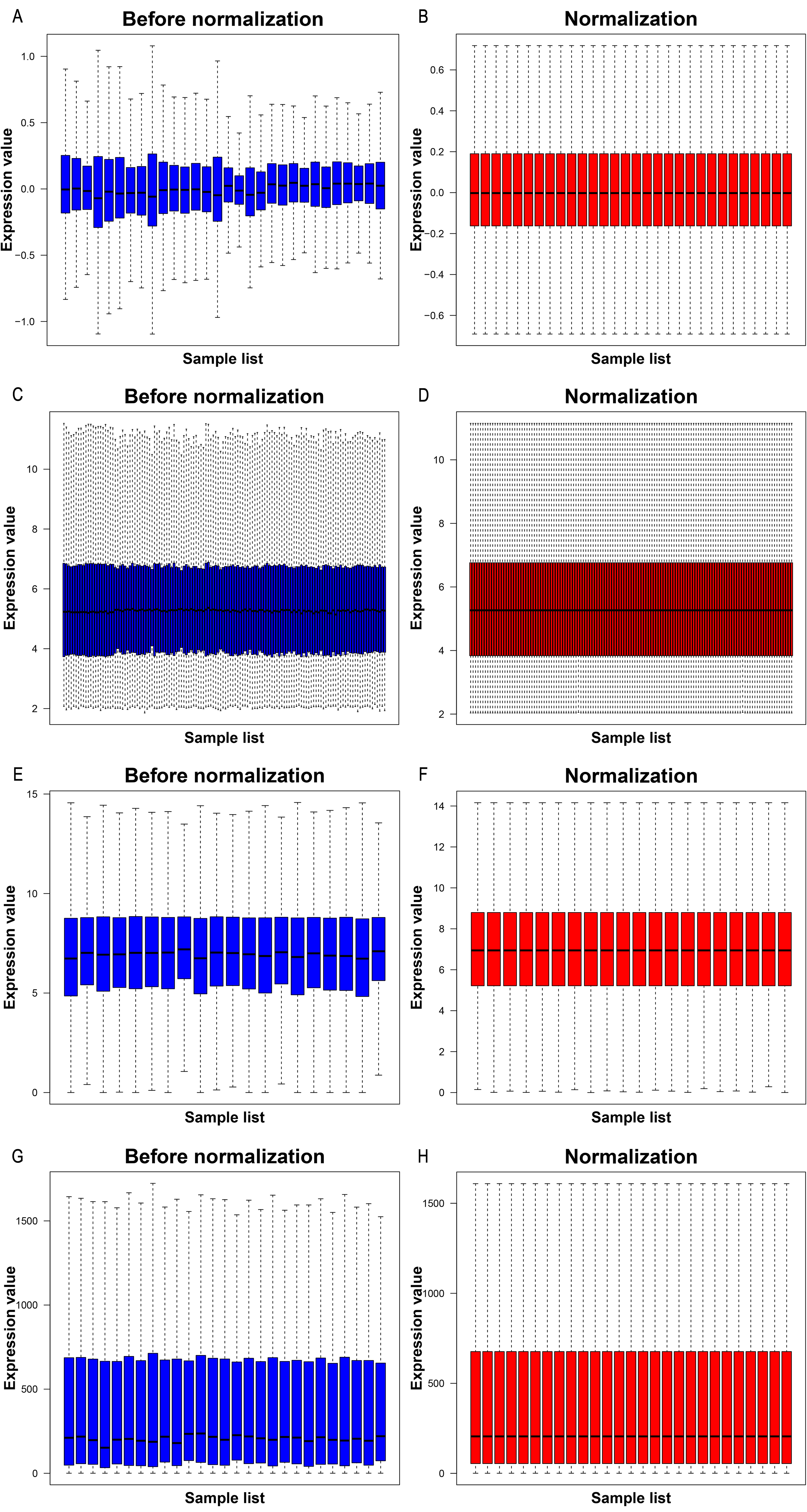


**Figure S1 Normalization of gene expression.** (A-B) Normalization of the GSE118916 dataset. (C-D) Normalization of the GSE54129 dataset. (E-F) Normalization of the GSE79973 dataset. (G-H) Normalization of the GSE19826 dataset. Blue represents data before normalization, and red represents data after normalization.

**Table S1 The integrated DEGs in gastric cancer**

| Gene | logFC | Gene | logFC | Gene | logFC | Gene | logFC | Gene | logFC |
| --- | --- | --- | --- | --- | --- | --- | --- | --- | --- |
| THBS2 | 3.463455 | ITGBL1 | 1.777912 | CHIA | -4.07864 | LDHD | -1.88507 | ALDH1A1 | -1.88076 |
| FAP | 3.610364 | SULF2 | 1.430232 | TMED6 | -3.40757 | UPK1B | -2.73767 | XK | -1.79475 |
| SFRP4 | 3.079173 | FKBP10 | 1.961738 | CWH43 | -3.53702 | AKR7A3 | -2.37967 | ANKRD22 | -1.62906 |
| SULF1 | 3.038803 | TMEM158 | 1.463692 | PGC | -3.56268 | PKIB | -2.62937 | MFSD4 | -2.93504 |
| FNDC1 | 3.585353 | MFAP2 | 1.986806 | FBP2 | -3.22261 | SULT1B1 | -2.12265 | AKR1C1 | -1.73182 |
| THBS4 | 2.855174 | MGP | 2.048681 | LIPF | -4.20796 | CAPN8 | -2.53345 | FOLR1 | -2.11363 |
| INHBA | 3.179046 | DPYSL3 | 1.825499 | SOSTDC1 | -4.16764 | ARL14 | -2.41951 | RAB27B | -1.99939 |
| RARRES1 | 2.578143 | COL3A1 | 1.49481 | ALDH3A1 | -3.06128 | MAP7D2 | -2.72797 | SPINK1 | -1.61111 |
| COL6A3 | 2.26181 | BGN | 2.049611 | UGT2B15 | -2.85761 | CNTN3 | -2.25079 | C1orf116 | -1.58602 |
| THY1 | 2.45748 | PLA2G2A | 2.459689 | PIK3C2G | -2.88152 | IRX3 | -2.39514 | KLHDC7A | -1.55956 |
| ASPN | 2.426879 | HOXB7 | 1.610325 | RDH12 | -2.76536 | PBLD | -2.09892 | FAM150B | -2.45186 |
| COL1A2 | 2.223111 | IL32 | 1.764056 | AKR1B10 | -3.58355 | KLK11 | -2.38499 | CNTD1 | -2.34508 |
| CXCL8 | 2.562077 | GREM1 | 2.288644 | HPGD | -2.94775 | HEPACAM2 | -2.19138 | RAB27A | -1.66316 |
| SPP1 | 2.800962 | CLDN3 | 1.743338 | ESRRG | -4.05826 | GC | -2.84601 | EPN3 | -1.63847 |
| TIMP1 | 2.064491 | PLAU | 1.368797 | CA2 | -2.62757 | ST6GALNAC1 | -1.80667 | ZNF57 | -1.5062 |
| CTHRC1 | 2.612272 | ISLR | 1.697335 | FAM3B | -2.84504 | CYP2C9 | -1.76133 | REG3A | -1.69391 |
| COL10A1 | 2.866131 | COL5A2 | 1.608804 | CTSE | -2.39997 | GPR64 | -2.60091 | AZGP1 | -2.53289 |
| FN1 | 2.104752 | HTRA1 | 1.397651 | ETNPPL | -3.46663 | PLAC8 | -1.91617 | GSTA1 | -1.74968 |
| PRRX1 | 1.930106 | LY6E | 2.071551 | ADTRP | -2.61045 | CAPN13 | -2.12348 | TMEM27 | -1.98581 |
| SFRP2 | 3.010099 | CXCL9 | 1.740192 | KRT20 | -4.18139 | SMIM24 | -1.89141 | TNFRSF17 | -1.92434 |
| CRISPLD1 | 2.305841 | LGALS1 | 1.476619 | SCNN1B | -2.36332 | SLC28A2 | -2.42002 | RNASE4 | -1.4906 |
| SERPINH1 | 2.380287 | TREM1 | 1.434313 | KCNJ16 | -3.36076 | MAL | -2.69804 | STX19 | -1.77693 |
| SPOCK1 | 2.431548 | FJX1 | 1.37141 | FUT9 | -2.87468 | MSMB | -2.98041 | LRRC31 | -1.82573 |
| OLFML2B | 1.925195 | EMILIN1 | 1.306295 | CLDN18 | -2.56088 | CYSTM1 | -1.70237 | AADAC | -2.44661 |
| SPARC | 1.805752 | COL8A1 | 2.021202 | C6orf58 | -2.94906 | SOWAHA | -1.69114 | APOBEC1 | -2.10211 |
| AEBP1 | 1.701319 | EDNRA | 1.556945 | ADH7 | -3.09352 | CLIC6 | -2.62942 | NEUROD1 | -1.59479 |
| NID2 | 1.816189 | CEMIP | 1.655568 | SLC26A9 | -2.93802 | MUC5AC | -2.37586 | SH3RF2 | -1.52956 |
| CST1 | 3.043291 | CHI3L1 | 2.121833 | TFF1 | -2.85317 | HRASLS2 | -2.08817 | SCGN | -1.62609 |
| MMP7 | 1.914676 | CEBPB | 1.267072 | C16orf89 | -2.68791 | KCNJ15 | -2.18342 | TMEM171 | -1.98216 |
| GUCY1A3 | 1.998938 | COL1A1 | 1.827244 | TCN1 | -2.43499 | ACKR4 | -2.14116 | CYP3A5 | -1.90577 |
| COL11A1 | 2.711257 | C3 | 2.441026 | LYPD6B | -2.68749 | FMO5 | -1.86079 | NOSTRIN | -1.35096 |
| CAP2 | 1.877671 | CDH3 | 2.161642 | ADH1C | -3.15106 | PLLP | -2.06074 | SELENBP1 | -1.48914 |
| CDH11 | 1.502078 | GIF | -5.651 | CYP2C18 | -2.69571 | CKMT2 | -2.50956 | TRIM50 | -2.18223 |
| COL5A1 | 1.590543 | GKN2 | -6.04348 | ATP4B | -3.92994 | DGKD | -1.63369 | FAM46C | -1.35651 |
| COL12A1 | 1.639078 | GKN1 | -5.92537 | REG1A | -2.60986 | DNER | -2.57748 | SULT2A1 | -2.15132 |
| CLDN1 | 2.12222 | DPCR1 | -4.5525 | DUOX2 | -2.37797 | FA2H | -1.53045 | ITPKA | -1.71023 |
| GPNMB | 1.987754 | VSIG1 | -4.14186 | PSAPL1 | -3.10177 | ANG | -1.78769 | LRRC66 | -2.24239 |
| CPXM1 | 2.03237 | ATP4A | -5.51597 | AQP4 | -3.11307 | MRAP2 | -1.82782 | PPFIBP2 | -1.27761 |
| FBN1 | 1.545245 | PSCA | -3.26246 | SSTR1 | -2.29839 | IL1R2 | -2.20088 | KIAA1324 | -1.98788 |
| VCAN | 1.549181 | TFF2 | -3.55171 | RASSF6 | -2.37063 | PDILT | -2.48654 | LIPH | -2.02617 |
| RAB31 | 1.785288 | KCNE2 | -4.78613 | SYTL5 | -2.06678 | AMPD1 | -2.07802 | NTN4 | -1.50542 |
| CLDN7 | 2.346942 | ANXA10 | -3.33517 | CPA2 | -3.43868 | PXMP2 | -1.79614 | MYZAP | -1.61025 |
| FAM83D | 1.541005 | SCGB2A1 | -3.73911 | SMIM6 | -2.59306 | PIGR | -1.65282 | BCAS1 | -2.37334 |
| PMEPA1 | 1.79721 | VSIG2 | -3.19734 | RFX6 | -2.46788 | COL2A1 | -2.57636 | GHRL | -2.5584 |
| HOXC6 | 2.44422 | CAPN9 | -3.46828 | PTPRZ1 | -2.20038 | HYAL1 | -1.70683 | GUCA2B | -2.32499 |
| COMP | 2.184175 | CA9 | -3.29913 | SULT1C2 | -2.77633 | MUM1L1 | -1.68982 | CCKBR | -2.52951 |
| PLEKHO1 | 1.777703 | CXCL17 | -3.57995 | LTF | -2.89768 | FCGBP | -2.58393 | DDX60 | -1.77955 |
| AHNAK2 | 1.633298 | SST | -4.17299 | CHGA | -3.27074 | LINC00261 | -2.30126 | SMIM5 | -1.76121 |

**DEGs,** Differentially expressed genes.

**Table S2 The KEGG analysis of the 14 genes**

| **Category** | **Term** | **Count** | **%** | **PValue** | **Genes** |
| --- | --- | --- | --- | --- | --- |
| KEGG_PATHWAY | hsa04512:ECM-receptor interaction | 7 | 0.310 | 2.80E-10 | *COL3A1, COL1A2, COL1A1, THBS2, COL5A2, COL5A1, FN1* |
| KEGG_PATHWAY | hsa04974:Protein digestion and absorption | 6 | 0.266 | 3.70E-08 | *COL3A1, COL1A2, COL12A1, COL1A1, COL5A2, COL5A1* |
| KEGG_PATHWAY | hsa04510:Focal adhesion | 7 | 0.310 | 5.23E-08 | *COL3A1, COL1A2, COL1A1, THBS2, COL5A2, COL5A1, FN1* |
| KEGG_PATHWAY | hsa05146:Amoebiasis | 6 | 0.266 | 9.48E-08 | *COL3A1, COL1A2, COL1A1, COL5A2, COL5A1, FN1* |
| KEGG_PATHWAY | hsa04151:PI3K-Akt signaling pathway | 7 | 0.310 | 1.13E-06 | *COL3A1, COL1A2, COL1A1, THBS2, COL5A2, COL5A1, FN1* |
| KEGG_PATHWAY | hsa04611:Platelet activation | 5 | 0.221 | 1.43E-05 | *COL3A1, COL1A2, COL1A1, COL5A2, COL5A1* |
